# chngpt: threshold regression model estimation and inference

**DOI:** 10.1186/s12859-017-1863-x

**Published:** 2017-10-16

**Authors:** Youyi Fong, Ying Huang, Peter B. Gilbert, Sallie R. Permar

**Affiliations:** 1 Department of Biostatistics, Bioinformatics and Epidemiology, Vaccine and Infectious Disease Division, Fred Hutchinson Cancer Research Center, USA, 1100 Fairview Ave N., Seattle, USA; 2 Human Vaccine Institute, Duke University Medical Center, 2 Genome Court, Durham, USA

**Keywords:** Segmented regression model, Regression kink, Jump-type, Change point

## Abstract

**Background:**

Threshold regression models are a diverse set of non-regular regression models that all depend on change points or thresholds. They provide a simple but elegant and interpretable way to model certain kinds of nonlinear relationships between the outcome and a predictor.

**Results:**

The R package *chngpt* provides both estimation and hypothesis testing functionalities for four common variants of threshold regression models. All allow for adjustment of additional covariates not subjected to thresholding. We demonstrate the consistency of the estimating procedures and the type 1 error rates of the testing procedures by Monte Carlo studies, and illustrate their practical uses using an example from the study of immune response biomarkers in the context of Mother-To-Child-Transmission of HIV-1 viruses.

**Conclusion:**

*chngpt* makes several unique contributions to the software for threshold regression models and will make these models more accessible to practitioners interested in modeling threshold effects.

**Electronic supplementary material:**

The online version of this article (doi:10.1186/s12859-017-1863-x) contains supplementary material, which is available to authorized users.

## Background

Threshold regression models are a class of regression models where the predictors are associated with the outcome in a threshold-dependent way. By introducing a threshold parameter, also known as the change point, threshold regression models provide a simple but elegant and interpretable way to model certain kinds of nonlinear relationships between the outcome and a predictor. There are many applications to threshold regression models in biomedical fields. Our interests in these models come primarily from the analyses of immunological assay data in human vaccine studies, where threshold-dependent association between risk of infection and immune response biomarkers abounds [1, 2].

**Fig. 1:**
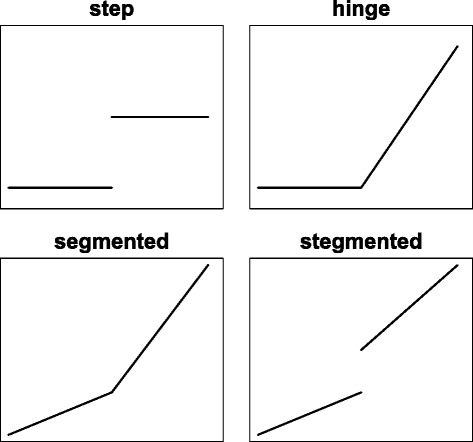
Four types of threshold effects

Threshold regression models can take many forms depending on what happens at the threshold [3]. For example, Fig. 1 shows four types of threshold effects: step, hinge, segmented and ‘stegmented’. The step and hinge models are two of the most basic forms of threshold effects with zero slope before the threshold; the segmented model generalizes the hinge model by allowing non-zero slope between the threshold; and the stegmented model, as the name suggests, can be viewed as the fusion of the step and segmented models.

In the generalized linear regression framework, we can write down the mean function of these four types of threshold models as follows:

$$\begin{array}{*{20}l} \eta & =\alpha_{1}+\alpha_{2}^{T}z+\beta_{1}I\left(x>e\right)\qquad\qquad\qquad\qquad\quad \text{step}\\ \eta & =\alpha_{1}+\alpha_{2}^{T}z+\beta_{1}\left(x-e\right)_{+}\qquad\qquad\qquad\qquad\,\,\text{hinge}\\ \eta & =\alpha_{1}+\alpha_{2}^{T}z+\beta_{1}\left(x-e\right)_{+}+\gamma x\qquad\qquad \text{segmented} \\ \eta & =\alpha_{1}+\alpha_{2}^{T}z+\beta_{1}\left(x-e\right)_{+}+\gamma x\\ &\quad+\beta_{2}I\left(x>e\right).\qquad\qquad\qquad\qquad\qquad \text{stegmented} \end{array} $$

Here *e* is the threshold parameter, *x* is the predictor with threshold effect, *z* denotes additional predictors, *I*(*x*>*e*)=1 when *x*>*e* and 0 otherwise, and (*x*−*e*)_+_ denotes the hinge function, which equals *x*−*e* when *x*>*e* and 0 otherwise.

Threshold regression models are related to but distinct from change-point analysis [4], which deal with time series data and are primarily concerned with detecting structural changes along a natural axis such as time or location on a chromosome. Many problems in change-point analysis are not regression problems. In change-point analysis regression problems, time series data are divided into regimens by change points; the relationships between the outcome and all the predictors are allowed to differ between regimens. In other words, all predictors are simultaneously thresholded in change-point analysis regression problems [5]. On the other hand, threshold regression models are fundamentally concerned with modeling nonlinearity. In this aspect, threshold regression models are more comparable to other nonlinear regression methods such as regression spline models [6].

Both threshold models and spline models are capable of modeling nonlinear relationships between the outcome and predictors. Their main differences lie in versatility and ease of interpretation. Take for example the hinge model and a natural cubic spline [7] with two degrees of freedom. Both have two degrees of freedom; in the case of the hinge model, the two relevant parameters are *β*_1_ and *e*. The spline model is more versatile than the hinge model, but when both models offer reasonably good fits, the hinge model is more readily interpretable [8].

While many software programs are available for change-point analysis and regression spline models, there are relatively few for threshold regression models. The best existing implementation is the R package *segmented* [5, 9], which supports the hinge and segmented models and allows multiple thresholds. Our *chngpt* package complements the *segmented* package by making three unique contributions: (1) it supports all four types of threshold effects in Fig. 1, and supports models with interaction terms between predictors subjected to thresholding and predictors not subjected to thresholding [10]; (2) the search method in *segmented* employs a first order approximation of the non-smooth criterion function [11], while *chngpt* offers two alternative search methods: exact, which optimizes the exact criterion function, and smooth, which approximates the criterion function with a logistic function-based smooth function [3]. The exact method guarantees to find the globally optimal solution but can be slow when the sample size is large, while the smooth method, like *segmented*, is faster but may find a locally optimal solution;

(3) *segmented* does not provide confidence intervals that account for the uncertainty of the threshold estimate, while *chngpt* do. The latter also includes model-robust confidence intervals, which are designed to provide proper coverage even if the data-generating model is not truly a threshold model [8].

## Implementation

### Estimation and confidence intervals

Estimation of threshold regression models is complicated by the fact that the models are not smooth in the threshold parameter. We consider two approaches for finding the maximum likelihood estimate of the threshold model parameters: *exact* and *smooth*. In the exact method we choose a grid of candidate change points that uniformly span the quantiles of the empirical distribution of the thresholded covariate. Given a candidate change point, the threshold regression model reduces to a regular regression model. The estimated change point is defined as the one corresponding to the reduced regression model with the highest likelihood. The advantages of the exact method are that it does not require a starting value for change point, and finds the global optimal solution; on the other hand, this method does not scale well to large datasets. As a practical measure, we set a default grid size of 500; when the sample size is larger than the grid size, we choose a subset of thresholded covariate values by uniformly sampling the ranks.

In the smooth method, we approximate the discontinuous likelihood function of a threshold regression model by approximating the step function *I*(*x*>*e*) with a two-parameter logistic function [1+ exp{*b*(*x*−*e*)}]^−1^ [3], where *b* is chosen to be an appropriately large constant. To find the parameter estimate, we perform iterated optimization. Given an initial estimate of all model parameters, we alternatively

1. update the threshold parameter *e* and the coefficients *β*’s and *γ* that are associated with thresholded covariate, conditional on the rest of the model parameters estimates, $\hat {\alpha }$,
2. update all coefficients, *α*, *β*’s and *γ*, conditional on the estimated threshold $\hat {e}$.

Due to approximation of the step function, the second step above has a smooth objective function and can be performed using a wide variety of optimization methods. Our choice is a quasi-Newton method with box constraint as implemented in the R function optim. We stop the algorithm when the relative changes dip below a pre-determined tolerance level. The main advantage of the smooth approximation algorithm is that even with a large dataset, it can converge relatively quickly. On the other hand, the solution found by this algorithm is a local optimum, and its performance depends critically on the choice of starting value. To get good starting values, we perform hypothesis testing on the coefficients associated with the thresholded covariate, which will be described next, and use the threshold value corresponding to the maximal test statistic as the starting value.

There are two basic types of confidence intervals for threshold regression model parameter estimates: model-based and model-robust. The former has the proper coverage only when the model is correctly specified, while the coverage of the latter is robust to model misspecification. There is an interesting interplay between these two types of confidence intervals and continuous (hinge and segmented) versus discontinuous (step and stegmented) threshold models. For continuous threshold models, all parameters converge at the regular *n* ^−1/2^ rate whether or not the model is correctly specified, but the asymptotic variance-covariance matrices differ [8, 12, 13]. For discontinuous threshold models, under correct model specification, the threshold estimate converges at a fast rate of *n* ^−1^, and the regression coefficient estimates converge at their usual rates of *n* ^−1/2^ [14]; under model misspecification, all parameters converge at the slower *n* ^−1/3^ rate [15]. In the *chngpt* package, we provide implementation of model-based intervals for all types of threshold models as well as model-robust confidence intervals for the continuous threshold models.

### Hypothesis testing

For hypothesis testing, we extend the methods in [10], which dealt with the step model, to handle all four types of threshold effects. In the step and hinge models, we are interested in testing the null hypothesis *β*_1_=0. In the segmented and stegmented models, the situation is more complex. We may be interested in testing all *β* and *γ* are 0, which corresponds to the null hypothesis that *x* has no effect on *Y*, or we may wish to leave *γ* out of the null hypothesis and test all *β* are 0. The latter puts the focus on threshold effect, and that is what we will focus on. Specifically, we will test *β*_1_=0 for the segmented model and *β*_1_=*β*_2_=0 for the stegmented model. When the null hypothesis involves only one parameter, either the maximum of scores or the maximum of likelihood ratios can be used. When the null hypothesis involves more than one parameter, tests based on maximum of likelihood ratios can be more powerful [10].

Consider the following equation that describes all threshold models in a uniform way:

$$\eta=\alpha^{T}\underline{z}+\beta^{T}\underline{x}\left(e\right), $$

where z represents all predictors independent of *e* and $\underline {x}\left (e\right)$ represents all predictors dependent on *e*. Let *p* be the dimension of $\underline {x}(e)$; *p*=2 in the stegmented model and *p*=1 otherwise.

Let *Q*(*e*) denote the likelihood ratio statistic for comparing a threshold model and the null model conditional on a candidate change point *e*. Assuming there are *M* candidate change points, the maximum of likelihood ratios test statistics is defined as

$${LR}_{\max}=\max\left\{ Q\left(e_{1}\right),\cdots,Q\left(e_{M}\right) \right\}. $$

Under the standard regularity conditions, *Q*(*e*) converges to a chi-squared distribution with *p* degrees of freedom under the null hypothesis. Furthermore, the statistic has the asymptotic representation

$$Q\left(e\right) =S_{\beta}^{T}\left(e\right) \hat{I}_{\beta\beta.\alpha }^{-1}\left(e\right) S_{\beta}\left(e\right) +o_{p}\left(1\right), $$

where $S_{\beta }^{T}\left (e\right) $ is the score vector for coefficients *β*, and $\hat {I}_{\beta \beta.\alpha }^{-1}\left (e\right) $ is the estimated information for *β* in the alternative model using $\hat {\mu }$ estimated under the null. In other words, *Q*(*e*) is asymptotically equivalent to the inner product of $S_{\beta }^{T}\left (e\right) \hat {I}_{\beta \beta.\alpha }^{-1/2}\left (e\right) $ and itself. Thus the joint distribution of {*Q*(*e*_1_),⋯,*Q*(*e*_*M*_)} can be estimated via a Monte Carlo procedure by simulating from a multivariate normal whose correlate matrix is the correlation matrix of $\{ S_{\beta }^{T}\left (e_{1}\right) \hat {I}_{\beta \beta.\alpha }^{-1/2}\left (e_{1}\right),$...,$S_{\beta }^{T}\left (e_{M}\right) \hat {I}_{\beta \beta.\alpha }^{-1/2}\left (e_{M}\right) \}^{T}$. Specifically, we

- Draw *B* independent random samples of size *pM* from a multivariate normal distribution with mean 0, variance 1 and correlation matrix derived from *JVJ*, where *J* is a block diagonal matrix with $\hat {I}_{\beta \beta.\alpha }^{-1/2}\left (e^{\ast }\right) $ on the diagonal and *V* is the variance-covariance matrix of $\left [ \begin {array} [c]{ccc} S\left (e_{1}\right) & \cdots & S\left (e_{M}\right) \end {array} \right ]^{T}$.
- Each of the *B* samples can be viewed as a sequence of *M* *p*-tuples of random variables. For the *b* ^*th*^ sample, compute the sum of squares for each *p*-tuple of random variables, and denote the maximum of the *M* sums of square by ${LR}_{\max }^{b}$.
- Obtain the p-value as $\#\left \{ {LR}_{\max }>{LR}_{\max }^{b}\right \} /B$.

## Results

### Monte Carlo studies

To validate the proposed methods and check the accuracy of the implementation, we conduct Monte Carlo experiments. We simulate data from logistic threshold regression models with true parameters listed in the Additional file 1: Table A.1. For the covariate distributions, we simulate from *z*∼*N*(0,*σ*=1), *x*∼*N*(4.7,*σ*=1.6). Each experiment is repeated 2,000 times to evaluate estimation performance and 10,000 times to evaluate hypothesis testing performance.

The results of estimation are collected in Tables 1 and 2. Table 1 shows the relative bias of the estimated coefficients and the bias of the estimated threshold. For the step, hinge, and segmented models, the relative bias is computed by dividing the difference between the Monte Carlo mean and the true value by the true value; for the stegmented model, the Monte Carlo mean is replaced by the Monte Carlo median due to the skewness of the distribution of the parameters. The results indicate that the estimates from both grid search and smooth approximation are asymptotically unbiased. We also see that the estimated coefficients associated with the thresholded covariate *x* have larger finite sample biases than the estimated coefficients associated with *z*, but the biases decrease as the sample size increases. Table 2 shows that the sampling variability decreases as the sample size increases as expected.

**Table 1:** Relative bias of coefficient estimates and bias of threshold estimates of three search strategies: grid search, smooth approximation, and first order approximation

	step		hinge		segmented		stegmented	
*n*	250	500	250	500	250	500	250	2000
grid								
z	0.02	0.02	0.02	0.02	0.02	0.00	0.04	0.01
x					0.22	0.09	0.48	0.02
I(x >e)	0.17	0.09					0.85	0.19
(*x*−*e*)_+_			0.15	0.05	0.23	0.11	-0.33	-0.05
e	0.00	-0.01	0.01	-0.02	0.01	0.03	-0.16	-0.02
smooth								
z	0.01	0.02	0.02	0.02	0.03	0.01	0.03	0.01
x					0.20	0.10	0.43	0.02
I(x >e)	0.13	0.06					0.35	0.03
(*x*−*e*)_+_			0.15	0.05	0.22	0.11	-0.26	-0.03
e	-0.02	-0.01	0.01	-0.02	0.03	0.01	-0.17	-0.03
first order								
z			0.02	0.02	0.02	0.00		
x					0.24	0.09		
(*x*−*e*)_+_			0.13	0.05	0.34	0.11		
e			-0.00	-0.02	0.04	0.04

**Table 2:** Monte Carlo interquartile range of coefficient and threshold estimates of three search strategies: grid search, smooth approximation, and first order approximation

	step		hinge		segmented		stegmented	
*n*	250	500	250	500	250	500	250	2000
grid								
z	0.19	0.14	0.19	0.14	0.20	0.15	0.22	0.07
x					0.40	0.26	0.68	0.17
I(x >e)	0.35	0.24					3.15	1.09
(*x*−*e*)_+_			0.50	0.31	0.49	0.31	1.19	0.24
e	0.31	0.15	0.69	0.45	1.00	0.65	1.47	0.57
smooth								
z	0.19	0.13	0.19	0.14	0.21	0.15	0.22	0.07
x					0.40	0.26	0.63	0.16
I(x >e)	0.35	0.24					2.84	0.97
(*x*−*e*)_+_			0.49	0.31	0.49	0.31	1.14	0.25
e	0.29	0.13	0.69	0.45	0.99	0.64	1.47	0.54
first order								
z			0.20	0.14	0.20	0.15		
x					0.41	0.26		
(*x*−*e*)_+_			0.49	0.31	0.53	0.31		
e			0.67	0.45	1.05	0.65		

In Tables 1 and 2 we also include the first order approximation search method that is available from the *segmented* package. These results show that the parameter estimates using this method have a similar profile to the estimates from the grid search and smooth approximation methods. An exception is when n=250, the estimated slope parameter associated with (*x*−*e*)_+_ in the segmented model is more biased by the first order approximation method, 0.34 versus 0.22 for grid search. To further compare their performance, we repeat this simulation study with a reduced magnitude of the slope parameter associated with (*x*−*e*)_+_ (*β*_1_=−0.51 instead of − 0.92). The results (Additional file 1: Table A.2 and A.3) show that the distance between the parameter estimates from the first order approximation and the grid search deviates increases dramatically, while the distance between the results from the smooth approximation and the grid search remains close.

Table 3 shows the type 1 error rates of hypothesis testing. We choose a moderate sample size of 250 for illustration. For the step, hinge, and segmented models, both the maximal score test and the maximal likelihood ratio test have close to nominal level type 1 error rates. For the stegmented model, the maximal likelihood ratio test type 1 error rate is slightly elevated, and as *p*=2, there is not a univariate score test that is directly comparable to the other three models.

**Table 3:** Type 1 error rates at sample size 250

	step	hinge	segmented	stegmented
LR	0.055	0.055	0.058	0.073
score	0.050	0.047	0.050	–

Finally, we compare the speed of grid search versus approximation methods. The model fitting is done on a Linux machine with a Intel^®;^ Xeon^®;^ CPU E5-2690 clocked at 2.90GHz. We examine four different samples sizes from 250 to 2000. The results from averaging over 10 simulations are shown in Table 4. For the smooth approximation method, model fitting for a dataset of 2000 rows takes less than one second, while for the exact method, model fitting for a dataset of 500 rows already takes more than one second. The performance of the first order approximation method also beats grid search but lags behind the smooth approximation method.

**Table 4:** Time (sec) for fitting threshold regression models

	grid					smooth					first order			
n	250	500	1000	2000		250	500	1000	2000		250	500	1000	2000
step	0.36	1.00	2.05	3.72		0.12	0.15	0.25	0.49					
hinge	0.37	1.01	2.09	3.77		0.11	0.15	0.25	0.49		0.30	0.40	0.60	1.09
segmented	0.39	1.07	2.14	3.97		0.12	0.16	0.27	0.55		0.25	0.38	0.65	1.34
stegmented	0.45	1.25	2.49	5.06		0.15	0.21	0.35	0.81					

### Real data illustrations

We illustrate the use of *chngpt* for fitting thresholded logistic regression models using a real data example from a study on the immunological biomarkers associated with the risk of Mother-To-Children Transmission (MTCT) of HIV-1 viruses [1]. The study was performed using stored blood samples from a cohort of U.S. non-breastfeeding, HIV-1–infected mother–infant pairs enrolled in the pre-ARV era Women and Infants Transmission Study [16]. Immunological assays were performed to measure antibody immune responses, including binding antibodies, neutralization antibodies, antibody avidity, and antibody-dependent cell-mediated cytotoxicity. The dataset includes 236 subjects, each corresponding to an infected mother; among them, 79 were transmitters and 157 are non-transmitters. For illustration, we consider the association between the transmission status and two covariates: *birth*, a categorical variable indicating the type of births, C-section or vaginal, and *NAb_SF162LS*, a continuous covariate giving the log titers of neutralization activities against a relatively easy to neutralize HIV-1 isolate named SF162LS.

**Fig. 2:**
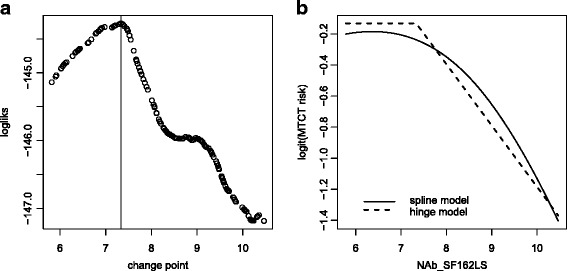
The HIV immune response and MTCT example. **a** Likelihoods of the restricted regression models with fixed change points versus candidate change points. **b** Predicted MTCT risks from a hinge model and a spline model

We fit the model using the exact search method, and a plot of the likelihood of the restricted regression model given a fixed change point versus the value of the change point is shown in Fig. 2 a. Figure 2 b plots the predicted risk as a function of *NAb_SF162LS* from the fitted model for vaginal births. For comparison, the figure also shows the predicted risk from a spline model that models the effect of *NAb_SF162LS* with a natural cubic spline with two degrees of freedom. Both the spline and hinge model fits suggest that the relationship between transmission and *NAb_SF162LS* is nonlinear; the hinge model fit further suggests that *NAb_SF162LS* needs to be above 7.4 (95% robust confidence interval 5.5, 8.2) before it is associated with decreased risk of MTCT.

To examine the classification performance of the hinge model, we perform Monte Carlo cross validation. We use 4/5 of the data to fit the model and a comparison model with *birth* only and use the rest of the data to evaluate classification performance of the models using the area under the ROC curve (AUC). Histograms of the AUC’s from 1000 repeats are shown in Additional file 1: Figure A.1. The median training and validation AUC of the *birth*-only model is 0.52 and 0.50, respectively; and the median training and validation AUC of the hinge model is 0.63 and 0.61, respectively. The Wilcoxon paired two-sample test comparing the validation AUC between the two models yield a p value of 2.2×10^−16^.

A second example illustrating the use of *chngpt* for fitting thresholded linear regression models can be found in the Additional file 1: Section B.

## Conclusions

We have developed an R package *chngpt* that supports four variants of threshold regression models that are most widely used in practice. The package implements both estimation and hypothesis testing functionalities based on a number of recent methodological advances [3, 8, 10]. *chngpt* is an open source software and can be downloaded from the Comprehensive R Archive Network. A short tutorial on how to use the package is contained in the Additional file 1: Section B.

Choosing among the four types of threshold models is an important and difficult question. We may divide this question into two parts: (i) whether a jump occurs at the threshold and (ii) whether the parameter space of the slope parameters should be restricted. The first question is especially challenging. For some processes, e.g. the occurrence of recombination events on a chromosome, it is natural to have jumps. For many others, the true underlying process may not be discontinuous; nonetheless, a discontinuous threshold model can be a useful approximation of a sudden shift in the response over a small span of the predictor values. To make this decision we recommend performing a test of the null hypothesis *β*_2_=0 based on the stegmented model using a method from [15]. For the second question, take the choice between the two continuous threshold models for example. The hinge model is nested within the segmented model, which may suggest that we always use the segmented model. However, as the simulation studies in [17] show, for the same sample size the hinge model can be estimated with much greater accuracy than the segmented model. Thus, if applicable, the hinge model would be preferred over the segmented model. The decision to use the hinge model should be based on a combination of statistical evidence, e.g. fitting the segmented model and checking whether the model-robust confidence interval of $\hat {\gamma }$ includes 0, and scientific consideration, e.g. if $\hat {\gamma }<0$ and yet, the predictor is not expected to have an inverse association with outcome given the domain knowledge, then it is more compelling to choose the hinge model when the confidence interval of $\hat {\gamma }$ includes 0.

## Additional file

Additional file 1 Supplementary tables and a short tutorial on using *chngpt*. (PDF 63.5 kb)

## Abbreviations

MTCT: Mother-To-Children Transmission
ARV: Antiretroviral drug

## References

[1] Permar SR, Fong Y, Vandergrift N, Fouda GG, Gilbert P, Parks R, Jaeger FH, Pollara J, Martelli A, Liebl BE, Lloyd K, Yates NL, Overman RG, Shen X, Whitaker K, Chen H, Pritchett J, Solomon E, Friberg E, Marshall DJ, Whitesides JF, Gurley TC, Holle TV, Martinez DR, Cai F, Kumar A, Xia SM, Lu X, Louzao R, Wilkes S, Datta S, Sarzotti-Kelsoe M, Liao HX, Ferrari G, Alam SM, Montefiori DC, Denny TN, Moody MA, Tomaras GD, Gao F, Haynes BF (2015). Maternal hiv-1 envelope–specific antibody responses and reduced risk of perinatal transmission. J Clin Investig.

[2] Moodie Z, Juraska M, Huang Y, Zhuang Y, Fong Y, Self SG, Chambonneau L, Small R, Jackson N, Noriega F, Gilbert PB. Neutralizing antibody correlates analysis of tetravalent dengue vaccine efficacy trials in asia and latin america. 2016. University of Washington, Technical report. 10.1093/infdis/jix609 PMC5854020 29194547

[3] Pastor-Barriuso R, Guallar E, Coresh J (2003). Transition models for change-point estimation in logistic regression. Stat Med.

[4] Chen J, Gupta AK (2011). Parametric Statistical Change Point Analysis: with Applications to Genetics, Medicine, and Finance.

[5] Muggeo VM (2008). Segmented: an r package to fit regression models with broken-line relationships. R news.

[6] Wakefield J (2012). Bayesian and Frequentist Regression Methods. Springer Series in Statistics Series.

[7] Hastie TJ, Tibshirani RJ (1990). Generalized Additive Models. Chapman and Hall/CRC Monographs on Statistics and Applied Probability Series.

[8] Fong Y, Chong D, Huang Y, Gilbert PB (2017). Model-robust inference for continuous threshold regression models. Biometrics.

[9] Muggeo VM (2016). Testing with a nuisance parameter present only under the alternative: a score-based approach with application to segmented modelling. J Stat Comput Simul.

[10] Fong Y, Di C, Permar S (2015). Change point testing in logistic regression models with interaction term. Stat Med.

[11] Muggeo VMR (2003). Estimating regression models with unknown break-points. Stat Med.

[12] Zhou H, Liang KY (2008). On estimating the change point in generalized linear models. IMS Collections Beyond Parametrics in Interdisciplinary Research: Festschrift in Honor of Professor Pranab K. Sen.

[13] Chan KS, Tsay RS (1998). Limiting properties of the least squares estimator of a continuous threshold autoregressive model. Biometrika.

[14] Hansen BE (2000). Sample splitting and threshold estimation. Econometrica.

[15] Banerjee M, McKeague IW (2007). Confidence sets for split points in decision trees. Ann Stat.

[16] Rich KC, Fowler MG, Mofenson LM, Abboud R, Pitt J, Diaz C, Hanson IC, Cooper E, Mendez H, Group ITS, for the Women (2000). Maternal and infant factors predicting disease progression in human immunodeficiency virus type 1-infected infants. Pediatrics.

[17] Fong Y, Chong D, Huang Y, Gilbert PB (2017). Model-robust inference for continuous threshold regression models. Biometrics.

