# Supplementary material for: chngpt: threshold regression model estimation and inference
Source: BMC Bioinformatics. 2017 Oct 16;18:454. doi: 10.1186/s12859-017-1863-x (PMC5644082; doi:10.1186/s12859-017-1863-x)
Supplement: Additional file 1 — Supplementary tables and a short tutorial on using chngpt. (PDF 63.5 kb) [file 12859_2017_1863_MOESM1_ESM.pdf]

Supplementary Materials for chngpt: threshold regression model estimation and inference

Youyi Fong, Ying Huang, Peter Gilbert, Sallie Permar

A Supplementary tables

	step	hinge	segmented	stegmented
z	0.34	0.34	0.34	0.34
x			0.40	-0.40
I(x>e)	-0.92			-0.40
$(x-e)_+$		-0.92	-0.92	-0.92
e	4.50	4.50	4.50	4.50

Table A.1: Monte Carlo experiment truth.

	step		hinge		segmented		stegmented	
n	250	500	250	500	250	500	250	2000
grid								
z	0.02	0.02	0.01	0.01	0.03	0.01	0.04	0.01
x					0.27	0.16	0.43	0.00
I(x>e)	0.46	0.28					1.32	0.22
$(x-e)_+$			0.34	0.13	0.48	0.30	-0.35	-0.07
e	0.05	0.01	0.02	-0.01	0.09	0.04	-0.02	-0.02
smooth								
z	0.01	0.02	0.01	0.01	0.03	0.02	0.05	0.01
x					0.25	0.18	0.42	0.00
I(x>e)	0.38	0.23					0.83	0.11
$(x-e)_+$			0.34	0.13	0.44	0.30	-0.21	-0.03
e	0.03	-0.02	0.01	-0.01	0.08	0.00	-0.01	-0.03
first order								
z			0.01	0.01	0.03	0.01		
x					1.27	0.22		
$(x-e)_+$			0.46	0.13	-22.36	-0.62		
e			0.04	-0.01	0.01	0.02		

Table A.2: Relative bias of coefficient estimates and bias of threshold estimates of three search strategies: grid search, smooth approximation, and first order approximation. Simulation setting same as in Table 1 except that β_1, the slope associated with $(x-e)_+$, is set to -0.51 instead of -0.92.

	step		hinge		segmented		stegmented	
n	250	500	250	500	250	500	250	2000
grid								
z	0.19	0.13	0.19	0.13	0.21	0.16	0.21	0.07
x					0.41	0.27	0.69	0.19
I(x>e)	0.36	0.25					2.98	0.97
$(x-e)_+$			0.44	0.27	0.51	0.33	1.21	0.23
e	0.97	0.51	1.28	0.81	1.80	1.28	1.81	0.66
smooth								
z	0.19	0.13	0.19	0.13	0.22	0.15	0.21	0.07
x					0.41	0.27	0.62	0.16
I(x>e)	0.37	0.25					2.68	0.79
$(x-e)_+$			0.44	0.27	0.52	0.33	1.16	0.23
e	0.96	0.50	1.27	0.82	1.87	1.25	1.82	0.59
first order								
z			0.19	0.13	0.21	0.16		
x					0.55	0.31		
$(x-e)_+$			0.47	0.27	0.73	0.39		
e			1.26	0.80	2.47	1.47		

Table A.3: Monte Carlo interquartile range of coefficient and threshold estimates of three search strategies: grid search, smooth approximation, and first order approximation. Simulation setting same as in Table 2 except that β_1, the slope associated with $(x-e)_+$, is set to -0.51 instead of -0.92.

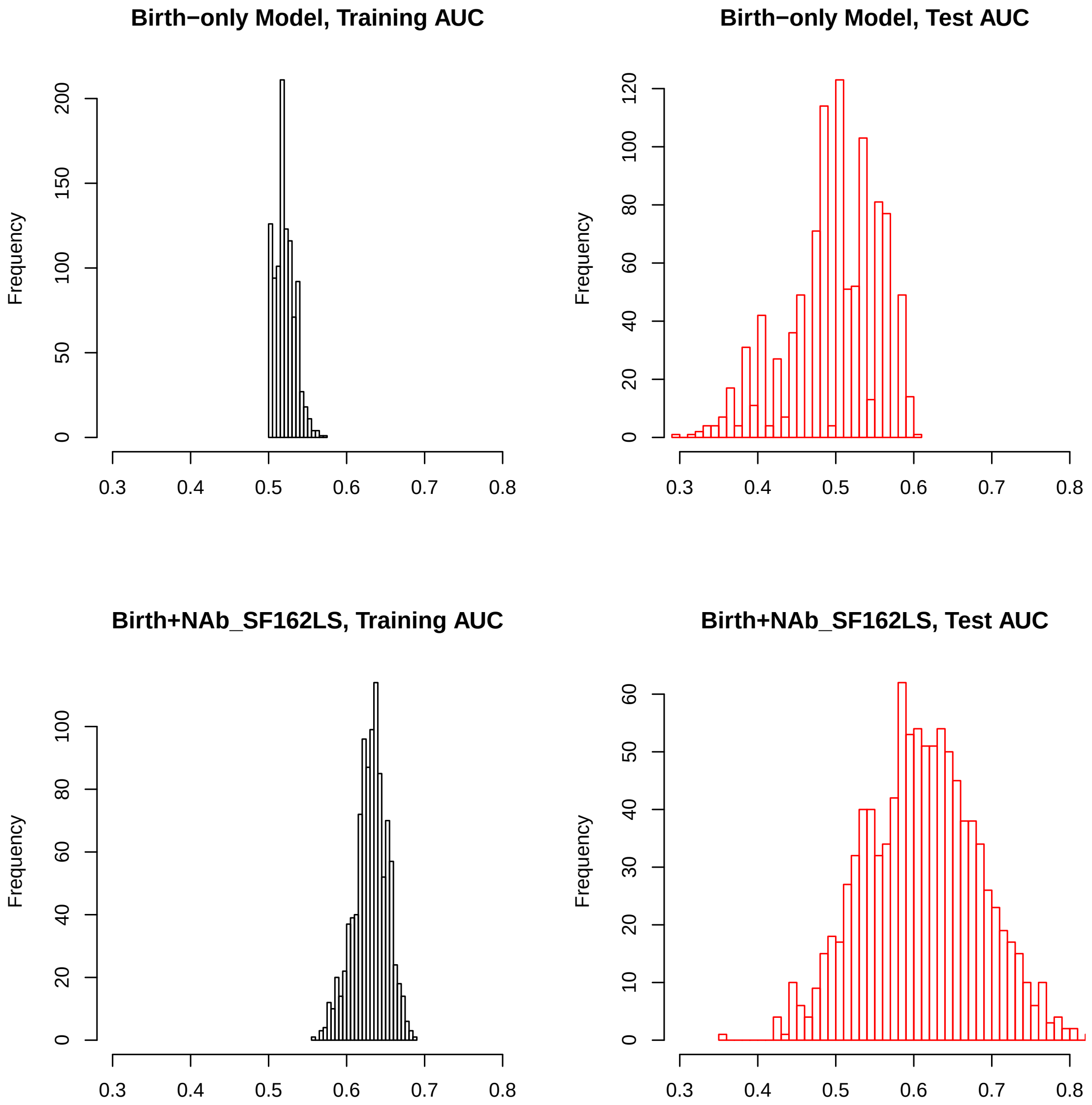

Figure A.1: Histograms of training and validation data AUC for the MTCT data example.

B *Trees* example

Our second example illustrates segmented linear regression for continuous outcome. The example uses the classic cherry trees dataset (Atkinson, 1987) that is part of the R *datasets* package. The dataset includes the girth and volume of 31 black berry trees. The nonlinearity in the relationship between volume and girth has been observed before, e.g. https://en.wikipedia.org /wiki/Multivariate_adaptive_regression_splines. We fit a segmented model using the exact search method. A plot of the likelihood of the restricted regression model given a fixed change point versus the value of the change point is shown in Supplementary Materials Figure B.1(a). Figure B.1(b) shows the fitted line and the observations. An interesting feature of this dataset is that there are two peaks in the likelihood versus change point curve (Figure B.1a). The *chngpt* package estimated the threshold to be 16.0 (95% bootstrap confidence interval 12.9, 18.0), which corresponds to the higher peak, while others have chosen the lower peak as the threshold estimate (https://en.wikipedia.org/wiki/ Multivariate_adaptive_regression_splines#The_basics).

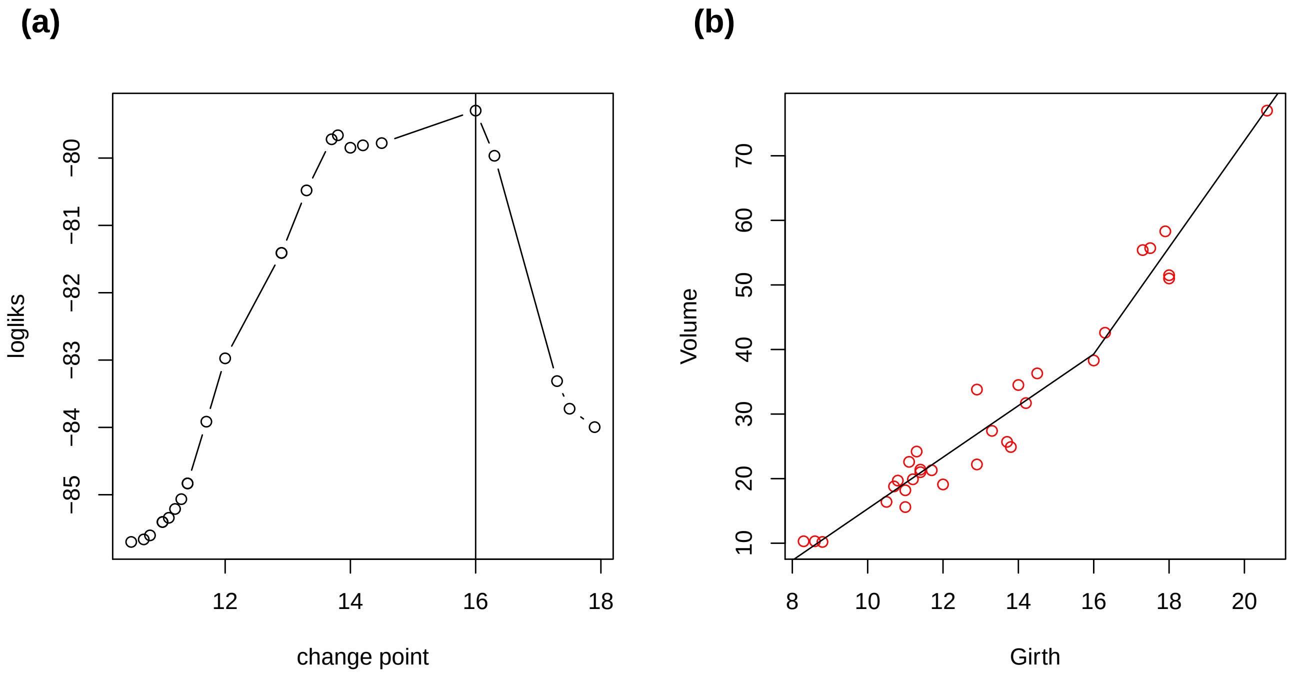

Figure B.1: The cherry trees example. (a) Likelihoods of the restricted regression models with fixed change points versus candidate change points. (b) The observations and the fitted line.

References

Atkinson, A. (1987), *Plots, Transformations, and Regression: An Introduction to Graphical Methods of Diagnostic Regression Analysis*, Oxford science publications, Clarendon Press, Oxford, UK.
